# Self-Optimizing Flow Reactors Get a Boost by Multitasking

**DOI:** 10.1021/acscentsci.3c00548

**Published:** 2023-05-08

**Authors:** Jason D. Williams, C. Oliver Kappe

**Affiliations:** Center for Continuous Flow Synthesis and Processing (CC FLOW), Research Center Pharmaceutical Engineering GmbH (RCPE), Inffeldgasse 13, Graz 8010, Austria; Institute of Chemistry, University of Graz, NAWI Graz, Heinrichstrasse 28, Graz 8010, Austria

**Figure 1:**
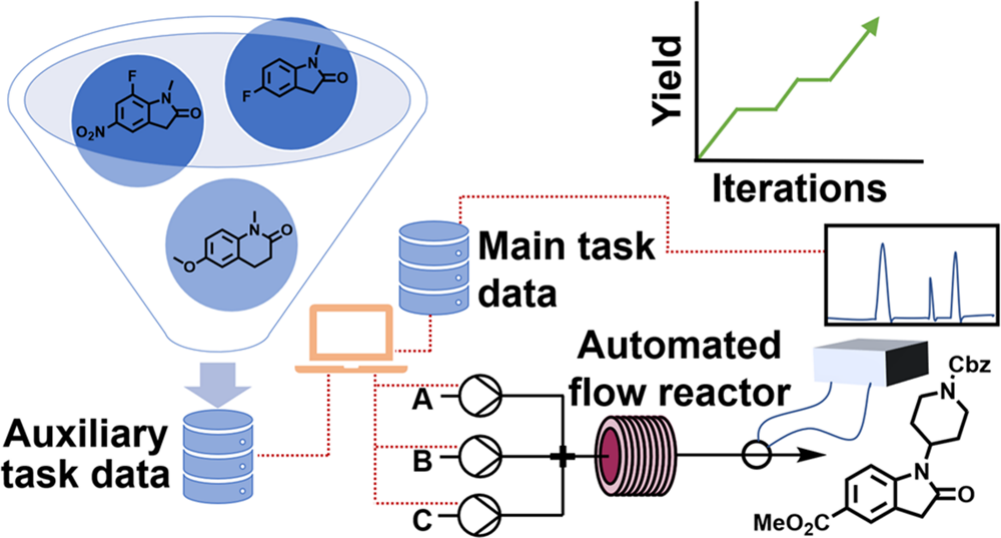
Schematic overview of the present work, which uses pre-existing data (auxiliary task) to assist in self-optimization in a flow reactor (main task)

In recent years, there has been a significant focus on both autonomous optimizations of organic reactions and the generation/use of large data sets of reaction results. However, there is still no clear “best approach” to reaction optimization. In this issue of *ACS Central Science*, Taylor, Lapkin, and co-workers, in a collaboration between Astex Pharmaceuticals and the University of Cambridge, combine the use of pre-existing data and self-optimization algorithms to the best effect.^1^ Their multitasking optimization algorithm (multi-task Bayesian optimization, MTBO) utilizes Bayesian optimization, which is generally viewed as the best algorithm type for “small data” reaction optimization, but simultaneously makes use of prerecorded reaction data within an auxiliary task. The algorithm was first disclosed in a 2021 preprint^2^ but was only demonstrated using *in silico* examples. This report provides the first validation of the method in the laboratory, for a genuine synthetic chemistry optimization problem (Figure 1).

The authors chose to make use of a flow reactor to demonstrate the MTBO algorithm. Although flow chemistry is often used to perform chemistries incompatible with batch processing methods (especially on a large scale), it also provides a platform for rapidly performing closed loop experimentation with relatively small quantities of material.^3^ While such iterative processes have also been reported using batch processing, flow chemistry allows a response to each individual experiment and is generally easier to automate. The use of a liquid handler to make up the reaction mixture also enabled this study to include categorical variables (e.g., solvent and ligand) in the optimization problem, a consideration that is very difficult to handle in standard optimization approaches.^4^

In the early days of self-optimizing flow reactors, the lack of requirement for any *a priori* reaction knowledge (e.g., mechanistic proposals and pre-existing data sets) was seen as a benefit. What could be better than getting optimum reaction conditions at the touch of a button, without having to do any prior research? Indeed, the curation and usage of pre-existing reaction sets can be cumbersome, but initiatives such as the Open Reaction Database^5^ and repositories such as Zenodo (operated by CERN) begin to make this task more manageable. In this report, the authors first make use of publicly available Suzuki coupling and Buchwald–Hartwig data sets for *in silico* demonstration, before moving on to demonstrating their experimental optimization.

One key finding in the *in silico* optimization was that the MTBO algorithm appears to function significantly better with a larger auxiliary task data set, particularly when multiple different substrates are present. This was put to good use in the Suzuki coupling case, which provided the best performance when all four available data sets were used for the auxiliary task. In general, one would assume that the more data that is available, the more efficient the optimization should be. Therefore, this naturally feeds back to a question that scientists, particularly in industry, have been trying to tackle for a number of years: how do we effectively record, curate, and utilize the results of past experiments? A recent editorial by scientists from AstraZeneca, University of Notre Dame, and MIT discusses this issue, particularly with regard to electronic laboratory notebooks (ELNs) and ensuring that *negative* data is effectively included.^6^

**Figure 2:**
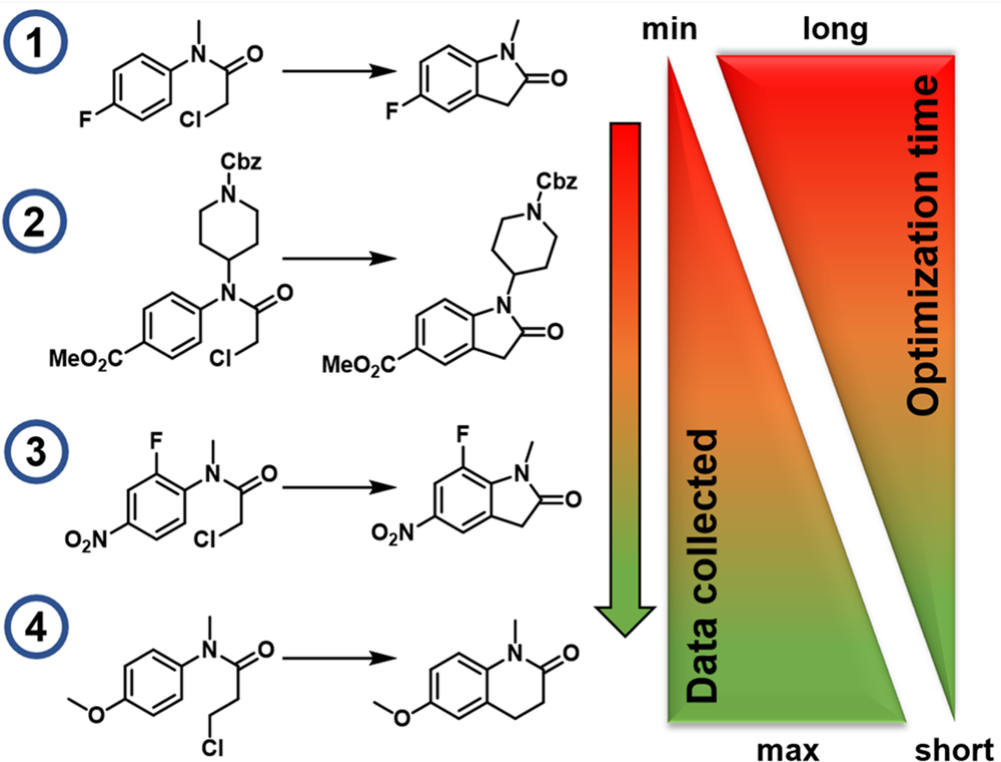
Substrates optimized in the present work, showing the increase in the quantity of data, and the corresponding decrease in optimization time with each substrate.

The chemistry used for laboratory demonstration was a C–H activation on small fragments with a relatively high proportion of polar functional groups, which can often be problematic in general synthetic methodologies. This is of significant importance, since molecules of interest in drug discovery are clearly applicable for such an approach. Here, as the auxiliary data set grew with each substrate, the rate of optimization was reported to increase (Figure 2), although this is difficult to quantify with substrates of differing reactivities. Within any research group, having access to such a living data set for commonly used reaction types could offer a huge advantage—reducing reliance on a chemist’s empirical experience of which conditions work best for certain substrate groups.

Now that MTBO is available for use by others (as part of Lapkin’s python-based Summit optimization package),^7^ we should start to see its true potential in the near future. This could include improvements on the currently presented setup such as using a droplet flow reactor, wherein small segments of the reaction mixture are separated with gas, to further decrease the consumption of precious catalyst and optimization materials.^8^ Another important consideration is the representation of categorical variables, which is achieved in the present report by simply assigning combinations of “1” and “0” to each categorical variable (known as one hot encoding, OHE). Other options, such as principal component analysis (PCA), could help to include information on the properties of these categorical variables, although the impact on optimization performance has not yet been shown to be significant.^9^ To take this one step further, in an approach similar to that previously demonstrated by Doyle and co-workers,^10^ can descriptors for reactants also be used to prioritize data from the most similar reaction partners within an auxiliary task data set? The possibilities are endless for this rapidly evolving field, but one thing is certain: as reaction optimization evolves away from the classical approach, organic chemists will continue to see new and effective options added to their optimization toolbox.
